# Controversial Roles of Regenerating Family Proteins in Tissue Repair and Tumor Development

**DOI:** 10.3390/biomedicines13010024

**Published:** 2024-12-26

**Authors:** Luting Yu, Qingyun Wu, Shenglong Jiang, Jia Liu, Junli Liu, Guoguang Chen

**Affiliations:** 1 School of Pharmaceutical Sciences, Nanjing Tech University, Nanjing 211816, China; lutingyu@njtech.edu.cn (L.Y.);; 2 MeDiC Program, The Research Institute of McGill University Health Centre, Montreal, QC H4A 3J1, Canada; 3 Division of Endocrinology and Metabolism, Department of Medicine, McGill University, Montreal, QC H4A 3J1, Canada

**Keywords:** regenerating family proteins, tissue repair, auto-immunogenicity, structure-function relationship, therapeutic agents

## Abstract

**Background**: Over the past 40 years since the discovery of regenerating family proteins (Reg proteins), numerous studies have highlighted their biological functions in promoting cell proliferation and resisting cell apoptosis, particularly in the regeneration and repair of pancreatic islets and exocrine glands. Successively, short peptides derived from Reg3δ and Reg3α have been employed in clinical trials, showing favorable therapeutic effects in patients with type I and type II diabetes. However, continued reports have been limited, presumably attributed to the potential side effects. **Methods**: This review summarizes extensive research on Reg proteins over the past decade, combined with our own related studies, proposing that Reg proteins exhibit dimorphic effects. **Results**: The activity of Reg proteins is not as simplistic as previously perceived but shows auto-immunogenicity depending on different pathophysiological microenvironments. The immunogenicity of Reg proteins could recruit immune cells leading to an anti-tumor effect. Such functional diversity is correlated with their structural characteristics: the N-terminal region contributes to autoantigenicity, while the C-type lectin fragment near the C-terminal determines the trophic action. It should be noted that B-cell masking antigens might also reside within the C-type lectin domain. **Conclusions**: Reg proteins have dual functional roles under various physiological and pathological conditions. These theoretical foundations facilitate the subsequent development of diagnostic reagents and therapeutic drugs targeting Reg proteins.

## 1. Introduction

Regenerating family protein (Reg) was first identified during studies of islet regeneration and cell proliferation. In 1984, Yonemura et al. found that Nicotinamide, an inhibitor of poly ADP-ribose polymerase (PARP), significantly promoted pancreatic expansion and islet regeneration in 90% pancreatectomized rats, supposing that this phenomenon was driven by a novel growth factor [1]. By screening the cDNA library, Terazono et al. uncovered the mysterious veil of this growth factor and nominated it as Reg [2]. It encoded the member of the Reg family protein 1 (Reg1), also known as pancreatic thread protein (PTP), pancreatic stone protein (PSP), and lithostathine [3,4], which consists of 165 amino acids with a signal peptide of 21 amino acids [2]. In 1999, Okamoto et al. systematically classified the *Reg* family genes in humans, rats, and mice into three subtypes, namely *Reg1*, *Reg2*, and *Reg3* [5]. *Reg4* was the last one to be identified and exhibited similar sequence homology, expression distribution, and intron-exon structure as other family members [6]. In 2000, Kobayashi et al. identified the receptor of rat Reg protein [7], which is homologous to human exostosin-like glycosyltransferase 3 (EXTL3) [8]. More recently, a new member of the Reg3 subfamily, Reg3E, was identified in the pancreas of dogs with acute pancreatitis [9,10].

Reg proteins exhibit a variety of biological activities and participate in numerous biological processes, as shown in Table 1. Research on the function of Reg proteins spotlighted their roles in pancreatic tissue repair and regeneration, particularly in the neogenesis of islet β cells. In vivo studies have shown that up-regulation of Reg family protein, induced by *IGF-1* gene deletion, had a protective effect in diabetic mice [11,12]. Reg1 (PTP, PSP), Reg3α (PAP), and islet regeneration-associated proteins (INGAP, Reg3δ) isolated from pancreatic acinar and ductal cells, respectively, could also stimulate the proliferation and regeneration of islet β cells [13,14,15,16]. INGAP-P, a short peptide fragment of 15 amino acids derived from Reg3δ, has demonstrated efficacy in the treatment of type I and II diabetes mellitus (T1DM and T2DM). As a candidate for anti-diabetic drug development, phase IIb clinical trials confirmed that INGAP-P significantly raised the area under the curve (AUC) of C-peptide in T1DM patients and reduced the hemoglobin A1C (HbA1c) levels in T2DM patients [13,17,18]. In 2014, a 12-week tolerability and safety study of Ustekinumab combined with INGAP-P was conducted in adult T1DM patients (https://trialsearch.who.int/Trial2.aspx?TrialID=NCT02204397 (accessed on 23 December 2024)), confirming that INGAP-P safely enhanced the viability of islet allografts post-transplantation. In summary, INGAP-P represents a novel class of antidiabetic therapeutic agents with the potential to address the underlying etiology of diabetes. However, in the past decade, clinical research on Reg family proteins and their derivatives has stalled, with no clear explanation for this stagnation. Notably, as research has progressed, the contradictory dimorphic effects of Reg proteins have become increasingly prominent [19,20,21]. This review examines the relevant studies on Reg proteins activity over the past decade, with a particular emphasis on resolving inconsistencies in the findings. It also comprehensively discusses the functions and mechanisms of Reg proteins under different physiological conditions. Additionally, the stagnation in clinical studies may also be related to potential side effects associated with Reg proteins.

## 2. Trophic Effect and Auto-Immunogenicity of Reg Protein

**Figure 1:**
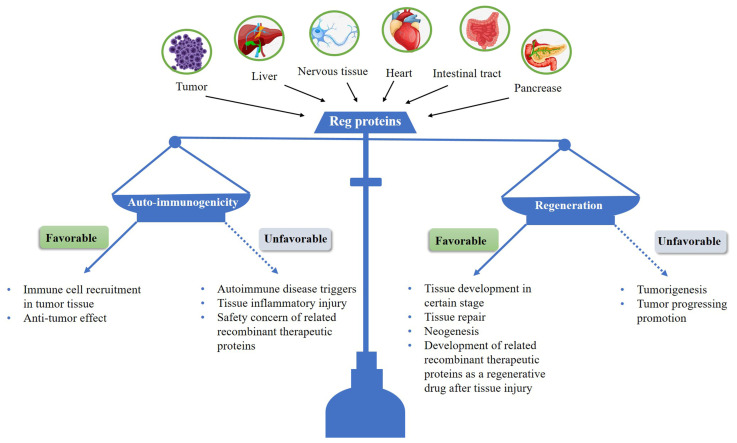
Contradictory dimorphic effects of regenerating family proteins. Reg proteins are highly expressed in the pancreas, intestinal tract, liver, heart, and nervous tissue, where they are thought to play a critical role in maintaining the balance between tissue regeneration and auto-immunogenicity. While their regenerative properties make them attractive candidates for applications in tissue repair and neogenesis, there is a risk of tumorigenesis. In parallel, the auto-immunogenic potential of Reg proteins may drive the development of autoimmune diseases and exacerbate inflammatory injury; however, this same mechanism could be crucial for their anti-tumor effects. Therefore, a comprehensive evaluation of these dual aspects is essential when assessing the druggability of Reg proteins, with the goal of optimizing their therapeutic potential while minimizing the adverse effects related to autoimmunity.

As a trophic factor, Reg proteins stimulate cell proliferation and facilitate repair through autocrine or paracrine manners in the pancreas, intestinal tract, liver, heart, or nervous tissue [22,23]. Generally, binding to its putative receptor EXTL3, Reg proteins activate PI3K-mediated Akt1/2 or Ras/Raf/Erk1/2 signaling pathways to promote phosphorylation of ATF-2, consequently up-regulating the expressions of Cyclin D1 and cyclin-dependent kinase 4 (CDK4) and inhibiting the expression of retinoblastoma protein (pRb) [15,16,24,25,26,27]. However, a few of the Reg family proteins have been confirmed to serve as autoantigens mediating inflammatory injury. They are involved in the occurrence and progression of autoimmune diseases including T1DM [28,29], lipoid-diarrhea [30], and primary Sjogren’s syndrome [31]. Thus, Reg proteins may play a crucial role in balancing tissue regeneration and autoimmunity (Figure 1), with dysregulation potentially leading to autoimmune injury and exacerbated inflammation.

### 2.1. Tissue Repair and Neogenesis Properties of Reg Proteins

#### 2.1.1. Pancreas

Reg family proteins are generally expressed in periods of embryonic development and pancreatic diseases, resulting in pancreas expansion and islet generation. The expression of Reg family proteins in embryonic stem cells was correlated with the activation of the WNT signaling pathway [32,33], and WNT/planar cell polarity (PCP) effector FLTP (also known as Flattop and Cfap126) played an important role in the maturation of β cell function [34]. Through modulating gene expression of pancreatic endocrine precursor markers Pdx1 and Ngn3, Reg3δ isolated from mice effectively reversed alloxan-induced diabetic hyperglycemia [35]. An oligopeptide derived from human Reg3A protein could also up-regulate the expression of pro-islet transcriptional factors to induce the differentiation of human fetus-derived pancreatic progenitor cells (HFPPCs) into functional β cells [36]. Reg1 protein in vesicles, which was released from acinar-like cell clusters touching Langerhans islets, contributed to β cell regeneration and restoration in encephalomyocarditis (EMC) virus-induced diabetic mice [37]. The CatL-E-cadherin-Reg pathway played a regulatory role in the proliferation of islet cells in response to signals generated by pancreatic exocrine tissue [38]. *Reg2* gene expression was required for appropriate islet compensation in response to obesity and aging [39]. Administration of recombinant Reg3α, Reg3β, or Reg3γ proteins in vivo could mitigate oxidative stress, preserve the number and function of islet β cells, and maintain glucose homeostasis [16,27,40,41]. Moreover, Reg proteins could reduce the protein folding burden and maintain cell function by up-regulating the expression of glucose-regulated proteins 78 (GRP78) chaperone [42] and activating the Erk1/2-STAT3 signaling pathway [40].

#### 2.1.2. Intestinal Tract

Besides being highly expressed in pancreatic tissue, Reg proteins were also found to be inducible in the intestinal tract [43,44], particularly Reg1 and Reg3 subfamily proteins that were induced by IL6/IL22 in inflammatory bowel disease (IBD) for mucosal regeneration through STAT3 activation [45,46,47,48]. Up-regulation of Reg3γ enhanced the physical and biochemical barrier function of the intestinal epithelium [49]. The anti-inflammatory proteins, Reg3β and Reg3γ, were induced in colitis to participate in the establishment of homeostatic equilibrium [50]. *Reg* genes regulated by GATA4 contributed to intestinal epithelial repair and intestinal microenvironment maintenance [51,52]. In addition, Reg3 subfamily proteins are also regarded as antimicrobial peptides, playing a crucial role in intestinal microbiota regulation and gut homeostasis. Activation of small intestinal Reg3A expression could attenuate intestinal bacterial overgrowth and translocation, abrogating hepatic inflammation along the gut–liver axis [53,54,55].

#### 2.1.3. Other Tissues

The reparative regeneration effect of Reg proteins is also exhibited in other tissues, such as the liver, heart, nerve, or skeleton. Reg3α and Reg3β induced in the associating liver partition and portal vein ligation for staged hepatectomy (ALPPS) activated the JAK2/STAT3 pathway and resulted in rapid liver regeneration [56], in accordance with the protective mechanisms in the pancreas. Increasing Reg protein expression induced by HMGB3 administration could improve cardiac function in myocardial ischemia [57,58]. It has also been shown that Reg2 facilitated nerve repair by attenuating neuroinflammation in experimental autoimmune encephalomyelitis [59,60], while Reg3γ was implicated in the regeneration of peripheral nerves and skeletal muscle [61]. Moreover, the expression of the *Reg* gene, stimulated by IL6, has been demonstrated to be crucial for the osteogenic response of the periosteum, which was pivotal for the fracture repair process [62].

### 2.2. Autoimmunity of Reg Proteins

#### 2.2.1. Pancreas

Although Reg3γ and Reg3β preserved β cells from autoimmune injury in T1DM by increasing regulatory T cell differentiation and inducing tolerated dendritic cells [63,64], the representative Reg2, highly expressed in islet cells and pancreatic ductal epithelial cells of non-obese diabetic (NOD) mice, has been identified as an important islet autoantigen inducing inflammatory responses and islet cell damage. Specific Reg2-reactive T cells extracted from lymph nodes of NOD mice were transfused into NOD mice of severe-combined immunodeficiency (SCID), resulting in the worsening of diabetes [28,65]. Furthermore, IFN-β could promote the expressions of *Reg1* and *Reg2* genes in NIT-1 cells by inducing the up-regulation of IL6 expression, and in NOD mice, its overexpression in islet β cells led to an increased Reg2 expression, thereby accelerating the deterioration of early diabetes [29]. Reg1 antibodies were also detectable in the serum of patients with T1DM [66]. Reg3α was also thought to be an autoimmune antigen that mediated islet damage, which was associated with the up-regulation of GRP78 [16,67]. In our early study, administration of recombinant Reg2 protein did cause obvious autoimmune reactions but would not trigger the onset of diabetes under normal conditions; however, a combination with STZ-caused islet β-cell damage and local inflammation Reg2-immunization aggravated the severity of diabetes [19]. Our recent data again indicated that Reg2-induced autoantibody posed a potential risk of accelerating diabetic progression [68].

#### 2.2.2. Other Tissues

In patients with primary Sjogren’s syndrome (SS), Reg1α induced by IL6/STAT pathway was detectable in salivary duct epithelial cells, and the salivary capacity was significantly decreased correlated with the presence of serum Reg1α autoantibody [31,69]. It is suggested that Reg1α-mediated autoimmune reaction causes damage and degeneration of salivary duct epithelial cells. Reg1α was also proved as a novel biomarker for diagnosis and prognosis of celiac disease in childhood-onset T1DM [70].

In addition, the action of Reg proteins has been observed to participate in certain pathological conditions that may be associated with immune activation. Reg proteins directly recruited inflammatory macrophage subsets but repressed that associated with healing and revascularization in the damaged heart tissue after myocardial infarction [71]. Reg-mediated signaling pathways might account for the activated inflammation in acute hepatic failure [72]. Both inflammation-related Reg3A and nerve injury-induced Reg2 acted as pro-inflammatory factors, individually contributing to the pathogenesis of diabetic retinopathy and the maintenance of neuropathic pain [73,74]. Contrary to the protective effect of intestinal mucosa, through altering gut microbiota and activating JAK2/STAT3 signaling pathway, Reg proteins promoted the recruitment of immune cells and intestinal inflammation [75,76]. PSP/Reg administration in animal models of sepsis exacerbated inflammatory response and the severity of multiorgan damage, which might be accomplished by promoting an inflammatory state of neutrophils [77].

## 3. Carcinogenesis and Tumor Suppression Effects of Reg Proteins

Reg proteins were initially identified in studies on islet cell proliferation and regeneration and were closely associated with pancreatitis and diabetes [16]. Notably, Reg protein also plays a significant role in the occurrence and progression of various cancers. Current research primarily focused on the detection of Reg3A and Reg4 levels in serum and tumor tissues, with superior accuracy and sensitivity compared to the existing biomarkers, making them promising novel indicators for both diagnosis and prognosis [78,79]. Nonetheless, the exact biological roles and mechanisms of Reg proteins in tumors remain controversial and need further investigation.

### 3.1. Role of Reg Proteins in Tumor Promotion

Recent studies have found that Reg proteins were highly associated with tumorigenesis and metastasis in pancreatic cancer [80,81,82], gastric cancer [83,84,85,86,87], colorectal cancer [88,89], head and neck cancer [90,91], ovarian cancer [92,93], and breast cancer [94]. Reg1α and Reg3β were highly expressed during acinar to-ductal metaplasia (ADM), with Reg3β promoting ADM formation through the MAPK signaling pathway. The expression levels of Reg1α and Reg1β were also positively correlated with pancreatic intraepithelial neoplasia (PanIN) [81]. Both ADM and PanIN could progress to invasive pancreatic ductal adenocarcinoma (PDAC). In gastric cancer, Reg1α promoted angiogenesis via the classical Akt and Erk signaling pathways [86], while Reg3A enhanced cell viability and drug resistance in ovarian cancer by activating the Akt signaling pathway [93]. In a mouse model of caerulein-induced chronic pancreatitis, Reg3γ overexpression accelerated pancreatic tumorigenesis, suggesting that Reg3γ aggravated inflammation and neoplastic progression [95]. This was consistent with the role of Reg3A in pancreatic cancer, where it drove tumor development via an IL6-JAK/STAT3-Reg positive feedback loop [80,96]. In colon cancer, high expression of Reg3A protein stimulated cell proliferation and tumorigenicity, resulting in larger tumor size, poor differentiation, and reduced survival rates [97,98]. In colorectal cancer, Reg4 expression, regulated by the transcription factor SP1, played an important role in triggering the EGFR/Akt/AP-1 signaling pathway [84,88,99]. Moreover, Reg4 contributed to 5-fluorouracil (5-FU) resistance in gastric cancer by modulating the MAPK/Erk/BIM signaling axis [100]. Additionally, Reg4 expression was up-regulated by caudal type homeobox 2 (CDX2), which further enhanced cancer cell migration and adhesion through the activation of SRY-related high-mobility group box 9 (SOX9) and G-protein-coupled receptor 37 (GPR37) [83,85,101]. These mechanisms collectively facilitate the peritoneal metastasis of gastric cancer cells.

### 3.2. Role of Reg Proteins in Tumor Suppression

Reg1α has been reported to enhance radiosensitivity in cancer cells by activating c-Jun expression, thereby increasing susceptibility to anti-tumor treatments [102]. Similarly, Reg3A inhibited the proliferation of gastric cancer cells via the PI3K/Akt-GSK3β pathway and promoted apoptosis by up-regulating DMBT1 [103,104]. In head and neck cancer cells, Reg3A improved sensitivity to chemotherapy and radiotherapy through the down-regulation of Cyclin D1 and BCL2 while activating the apoptotic factor Caspase3 [105]. In patients with multiple types of tumors, high expression of Reg3A was correlated with improved overall survival (OS) and disease-free survival (DFS) [104,106]. Additionally, endogenous overexpression of Reg3A enhanced T-cell infiltration, contributing to tumor suppression [20]. Furthermore, Reg4 expression in non-mucinous colorectal cancer served as an independent marker of favorable prognosis [107], which was completely contrary to our understanding of its tumor-promoting activity. A rational, mechanistic explanation for the conflicting roles of Reg family protein becomes increasingly urgent and essential.

## 4. Structure-Based Activity Inversion in Reg Proteins

**Figure 2:**
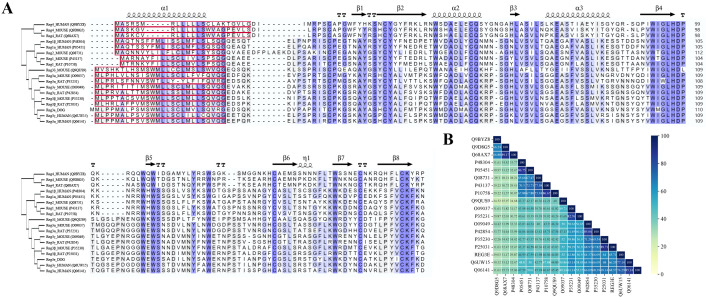
Sequence alignment of Reg family proteins. (**A**) Amino acid sequence alignment and secondary structure distribution of Reg protein family members. The intensity of the background color correlates with the degree of amino acid sequence similarity, with darker shades indicating higher similarity. The region enclosed by the red box corresponds to the signaling peptide region. (**B**) Similarity of sequence alignment in (**A**).

The primary sequences of Reg family proteins are highly homologic (Figure 2). They are composed of 160–170 amino acids, with a calcium-dependent lectin (C-type lectin) domain of about 120 amino acids containing a carbohydrate recognition site related to the processes of endocytosis, cellular immunity, and humoral immunity [108,109]. The N-terminal contains a signal peptide with more than 20 amino acids that mediates paracrine and autocrine secretion into an extracellular matrix [24,110]. There is a conserved sequence of 28 amino acid residues in Reg family proteins, including six cysteines, forming three pairs of intramolecular disulfide bonds [5], resulting in similar spatial structures of various Reg family molecules. Human and mouse Reg proteins have the same chromosomal location (except Reg4, solely located on 1p12-p21, whereas other protein-coding genes are located on 2p12), tandem mode on chromosomes, and intron–exon component connectivity, indicating that human and mouse Reg family genes have evolved from the same progenitor genes.

**Figure 3:**
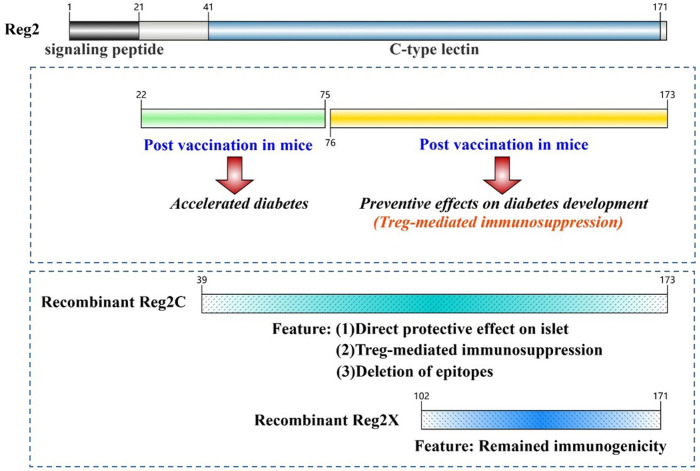
Bioactivity analysis of the Reg2 protein based on its structural feature. The amino acid sequence from residue 22 to 75 includes T-cell epitopes, while the region spanning residue 76 to 173 comprises Treg-activating domains. The C-type lectin domain is located between residue 41 and 171. Major B-cell epitopes are found within residues 22 to 41, with secondary B-cell antigenic epitopes present from positions 102 to 171. Deleting amino acids between residues 42 and 101 would expose the secondary B-cell antigenic epitopes, thereby restoring the immunogenicity of the Reg2X protein. The protein sequence in this figure was illustrated and presented using the online tool IBS 2.0 [115].

The Reg2 protein in mice and hamsters exhibits a distinct motif (QVAEEDE) near the N-terminus compared to other family members of Reg proteins, while all Reg3 proteins share a conserved sequence in this region, encompassing five amino acids (PNGGG) [111]. The sequential variation in the N-terminal residue may influence the autoantigenicity, whereas the highly conserved C-terminal region is primarily responsible for the biological function [28,112,113]. Previous studies have reported that vaccination with the N-terminal segment of Reg2 caused deterioration of diabetic conditions, whereas the C-terminal region provided protective effects [28]. Moreover, Reg1α promoted the differentiation of cortical progenitor cells through its N-terminal active domain [114], also suggesting that N-terminal and C-terminal domains may have distinct and independent functions. According to antigenicity prediction on Reg2 protein using the IEDB analytic tool (http://tools.iedb.org/main/ (accessed on 23 December 2024)), the Reg2C fragment (Ala 39 to Ala 173 of Reg2), which excluded the N-terminal residue but retained the conserved C-type lectin domain, dramatically reduced autoantigenicity while maintaining trophic bioactivity; in contrast, the Reg2X fragment (Ala 102 to Phe 171 of Reg2), which contained a dominant autoantigenic region and a carbohydrate-binding site (Gln 114 to Asn 116), stimulated cell proliferation and protected against apoptosis but exhibited strong immunogenicity [68]. The current findings offer a theoretical basis for optimizing protein sequences by eliminating immunogenic elements while preserving the desired biological activity (Figure 3).

Therefore, when both active regions are present simultaneously, the Reg proteins may exhibit markedly different roles under varying pathophysiological conditions. Indeed, while Reg2 did not cause immune damage in normal mice and even demonstrated islet-protective activity, in BALB/c mice hypersensitive to Reg2, there was a significant increase in the frequency of CD8+ T cells infiltration in the islets, leading to a reduced tolerance to STZ [19]. This suggests a potential risk associated with long-term administration of Reg2 in patients who are immunoreactive to it, particularly those with Reg2 autoantibodies or Reg2-reactive auto-aggressive T cells. Additionally, differences in protease activity within the tissue microenvironment could alter the fibro-genic capacity of Reg1α after cleavage [116], indicating that the activity of Reg proteins is influenced by both their structural configuration and external factors. Overall, when elucidating the biochemical function of Reg proteins and their role in drug development, it is essential to consider the tissue microenvironment and the unique structural characteristics of the Reg proteins. These factors are crucial in determining which of their dual roles predominates under specific conditions.

## 5. The Applications and Prospects of Reg Proteins

### 5.1. Reg Proteins as Novel Biomarkers

Reg proteins are predominantly expressed during embryonic development and gastrointestinal tissues in adults. Under certain pathological conditions, its expression levels may be significantly elevated, indicating its potential utility as a biomarker for the diagnosis and prognosis of related diseases. Here, we systematically compiled a list of potential physiological and pathological conditions where Reg proteins may serve as a novel biomarker (Table 2) based on research from the past decade, including the corresponding disease types and detection sites, to support further research and clinical applications.

#### 5.1.1. Infection

Recent studies have demonstrated a significant correlation between Reg proteins and infectious diseases. A systematic review and individual patient-level meta-analysis suggested that pancreatic stone protein/regenerating protein (PSP/Reg, also regarded as lithostathine or Reg1) was a promising biomarker for diagnosing infections and predicting infection severity and ICU mortality, outperforming C-reactive protein (CRP) or procalcitonin (PCT) [122,123]. In sepsis, one of the most severe infectious diseases, serum levels of Reg1 and Reg3 were significantly elevated [124,130,134,135], even in cases of sepsis-related multiorgan failure [77]. Reg proteins thus have substantial value for assessing disease severity and predicting prognosis in both adults and children with sepsis.

#### 5.1.2. Tumor

The expression of Reg protein in serum and tissue may also serve as a valuable biomarker for the diagnosis, classification, and prognosis of various malignant tumors. Reg1A has been identified as a predictive marker for the recurrence of urothelial tumors [128]. Serum concentrations of Reg1α and Reg1β have shown potential as early diagnostic indicators for PDAC and might also be useful in assessing therapeutic responses and predicting patient outcomes [81]. Additionally, Reg3A overexpression has been associated with reduced effectiveness of concurrent chemoradiotherapy and poor survival rates in patients with rectal cancer [89]. Notably, Reg4 exhibited higher diagnostic accuracy in pancreatic cancer compared to other family members [133].

#### 5.1.3. Other Diseases

Circulating Reg1 levels were elevated in patients with T2DM [117,136], chronic endoplasmic reticulum (ER) stress in β cells [118], and pregnancy-related diseases such as preeclampsia (PE), hemolysis-elevated liver enzymes, and low platelet (HELLP) syndrome [125]. The increases in circulating PSP/Reg in T2DM and pregnancy were also significantly associated with renal damage and dysfunction [119,120,121]. Reg3α has emerged as a novel biomarker linked to the site of graft-versus-host disease (GVDH) or tissue injury [131]. Additionally, Reg4 was not only highly expressed in various tumors but also up-regulated in IBD and intermittent myocardial hypoxia [47,58].

### 5.2. Reg Proteins as Therapeutic Targets

Studies have shown that the endogenous Reg proteins under physiological and pathological conditions participate in tissue repair and regeneration, suggesting the potential development of recombinant Reg proteins or agonists as novel therapeutic agents for disease prevention and treatment. Given the absence of glycosylation sites in Reg proteins, we initially employed a prokaryotic expression system to produce recombinant Reg family proteins (rRegs) and their derived peptides for therapeutic activity evaluation in pancreatitis and diabetes models. Among them, rReg3α and rReg4 demonstrated the ability to mitigate acute injury in severe pancreatitis by significantly reducing cell necrosis [137]. rReg2, rReg3α, and rReg3β exhibited protective effects on pancreatic islet and enhanced islet regeneration, indicating their potential in diabetes treatment [16,19,27,68]. Therefore, Reg protein-based therapies hold considerable promise for medicinal research. Further investigation is warranted to fully explore the therapeutic potential of Reg family proteins, particularly in balancing their treatment efficacy and immunological effects, with the goal of advancing them into effective therapeutic agents.

However, Reg proteins also play a negative role in certain diseases, where they can accelerate pathological processes. For instance, Reg1α was implicated in the progression of tauopathies, a class of neurodegenerative diseases, by sequentially phosphorylating tau proteins [138]. Similarly, Reg2 decreased the activities of superoxide dismutase-1 (SOD1) and glutathione peroxidase-1 (GPX1), thereby reducing cell viability in both murine pancreatic islets and human pancreatic cells [139]. Additionally, the overexpression of Reg3β, specifically in the pancreatic β-cell, has been associated with accelerated hyperglycemia and the worsening of T2DM in mice [140]. Moreover, a deficiency in the *Reg1–3* genes has been demonstrated to induce remission in chronic pancreatitis [141], further supporting the idea that Reg proteins might serve as promising therapeutic targets for various conditions. Given the pathogenic roles of Reg proteins, several therapeutic strategies can be developed to mitigate their detrimental effects. The first therapeutic strategy is antibody-based therapy, which aims to block the biological activity of Reg proteins. In our preliminary studies, the single-chain variable fragment (scFv) against Reg3A and Reg4, two of the most commonly overexpressed Reg proteins in tumors, have demonstrated significant anti-tumor effects [142,143]. The second therapeutic strategy involves the use of inhibitors. Eckol, for example, has been shown to inhibit Reg3A-induced proliferation in human pancreatic cancer cells [144], effectively suppressing downstream signaling pathways. Additionally, Reg4 has been identified as a therapeutic target in ovarian mucinous carcinoma, where its expression is modulated by CDX2 and has been associated with resistance to 5-FU-based chemotherapy [88,95]. The third therapeutic strategy is gene therapy, aimed at reducing the endogenous expression of Reg proteins. Knockdown of *Reg1α* has been shown to enhance the sensitivity of colorectal cancer cells to 5-FU [145], improving the efficacy of existing treatments. Similarly, Reg3A has been identified as a promising target for triple-negative breast cancer (TNBC). In both in vivo and in vitro studies, knockout of Reg3A has significantly suppressed tumor growth [94], highlighting its therapeutic potential.

## 6. Conclusions

This article provides a comprehensive review of research on Reg proteins over the past decade, emphasizing their dual functional roles under various physiological and pathological conditions, specifically in tissue repair and regeneration, as well as their auto-immunogenic properties. The observed stagnation of clinical trials using Reg proteins for islet regeneration therapy in diabetes is likely related to the side effects stemming from their auto-immunogenicity. Structural analysis of Reg proteins has identified the specific antigenic epitopes responsible for this auto-immunogenicity. Future research on structural modification of Reg proteins is needed to reduce their immunogenicity while maintaining therapeutic potential in tissue regeneration. In addition to their roles in normal tissues, Reg proteins have significant implications in tumor biology, contributing to tumorigenesis and metastasis. Interestingly, recent studies have revealed that Reg proteins exhibit tumor-suppressive effects in certain cancer subtypes. This paradoxical function, combined with their immunogenic properties, suggests that overexpressed Reg proteins can recruit immune cells to the tumor microenvironment, thereby exerting a degree of anti-tumor activity. Finally, the prospective applications of Reg proteins have been thoroughly explored and anticipated. Their aberrant overexpression in various diseases, including cancer and inflammation, positions them as potential biomarkers for disease diagnosis and prognosis. Notably, their abnormal expression in sepsis could establish Reg proteins as novel biomarkers for this life-threatening condition. Therapeutically, under the premise of a clear consensus on the biological function and mechanism of Reg proteins, there are two potential avenues: first, leveraging the tissue repair capacity of Reg protein by developing agonists or recombinant protein drugs, with careful removal of immunogenic elements; second, addressing their role in promoting tumor progression and autoimmune disorders by designing antibodies, inhibitors, or gene therapies to mitigate its detrimental effects. Although the controversial roles of Reg proteins have indeed captured our interest, a thorough elucidation of the precise underlying molecular mechanisms is imperative for future research. It includes clarifying the activation of signaling pathways by endogenous regulation of Reg expression or exogenous Reg administration. These insights are essential for deepening our understanding of Reg proteins’ bioactivity and optimizing their potential in drug development. Addressing the challenges related to their auto-immunogenic properties and capitalizing on their diverse functionalities will be crucial for integrating Reg proteins into advanced therapeutic strategies in regenerative medicine and oncology.

## Figures and Tables

**Table 1:** The biological effects of Reg family proteins.

Biological Effects	Affected Tissues or Organs	Involved Biological Processes	Involved Family Members
Proliferation-promoting	Pancreas, liver, GI, nerve, brain, skin, tumor	Mitogenic process, cell proliferation, tissue regeneration (neogenesis and injury repair), tumorigenesis and progression	All family members
Anti-apoptosis and anti-necrosis	Pancreas, liver, GI, nerve, brain, skin, tumor	Injury repair, anti-inflammatory response, tumor promotion	All family members
Antibacterial activity	GI	Modulating gut microbiota, maintaining gut homeostasis	Reg3 subfamily members
Pro-metastatic effects	Tumor	Tumor metastasis	Reg1, Reg3, Reg4
Immunogenicity	Pancreas, GI, salivary glands	The onset of autoimmune diseases	Reg1, Reg2, Reg3

**Table 2:** Potential physiological and pathological conditions for Reg proteins as novel biomarkers.

Type	Disease	Detection Site	References
Reg1	T2DM	Serum	[117,118]
Reg1	Diabetic kidney disease	Serum	[119,120,121]
Reg1	Infection, especially in emergency rooms (ER), burn and intensive care units (ICUs)	Whole blood	[122,123]
Reg1	Sepsis-related multiorgan failure and mortality	Serum	[77,124]
Reg1	Preterm premature rupture of membranes (PPROM), preeclampsia (PE)	Peripheral blood	[125]
Reg1	Patients with stage-IV head and neck squamous cell carcinoma	Biopsy specimens	[91]
Reg1	Inflammatory bowel disease	Biopsy specimens	[46]
Reg1α	Sepsis	Plasma	[126]
Reg1α	Pancreatic cancer	Ductal fluid	[127]
Reg1α	Superficial urothelial urinary bladder carcinomas	Histologic section	[128]
Reg1α	Gastrointestinal cancers, breast cancer, pulmonary cancer	Serum	[129]
Reg1α	Pancreatic adenocarcinoma	Ductal fluid	[127]
Reg1α	Celiac disease	Blood	[70]
Reg1β	Pancreatic adenocarcinoma	Ductal fluid	[127]
Reg3α	Pancreatic adenocarcinoma	Ductal fluid	[127]
Reg3α	Ovarian cancer	Tumor tissue	[93]
Reg3α	Rectal cancer	Histologic section	[89]
Reg3α	Triple negative breast cancer	Histologic section	[94]
Reg3α	Sepsis	Serum	[130]
Reg3α	GVHD or tissue injury	Plasma	[131]
Reg3α	Diabetic retinopathy	Aqueous humor	[73]
Reg3α	Transplant-related mortality	Serum	[132]
Reg4	Gastric cancer	Histologic section	[83,85]
Reg4	Pancreatic cancer	N/A	[133]
Reg4	Inflammatory bowel disease	Biopsy specimens	[46]

## Data Availability

Data availability is not applicable to this article as no new data were created or analyzed in this study.
